# Immune therapy of metastatic melanoma developing after allogeneic bone marrow transplant

**DOI:** 10.1186/s40425-015-0054-4

**Published:** 2015-03-24

**Authors:** Michael Cecchini, Mario Sznol, Stuart Seropian

**Affiliations:** Division of Medical Oncology, Yale Cancer Center, Smilow Cancer Hospital of the Yale-New Haven Hospital Yale University School of Medicine, New Haven, USA

**Keywords:** Melanoma, Immunotherapy, Interleukin-2, Graft vs host disease, Allogeneic transplant, Ipilimumab, CTLA-4 antigen, Programmed cell death 1 receptor and immune-related adverse events

## Abstract

Metastatic melanoma is frequently treated with immune activating therapy, which poses a theoretical risk of inducing graft versus host disease (GVHD) in those who have received allogeneic stem cell transplantation. The literature reporting the safety of immunotherapy in post transplant patients is limited. We report two patients with metastatic melanoma who received treatment with immunotherapy after allogeneic stem cell transplantation that did not result in GVHD.

## Background

The risk for developing a second malignancy is increased in long term survivors of allogeneic stem cell transplants (ASCT). Melanoma is one of the solid tumors observed at increased frequency in this setting, and can progress to metastatic disease requiring systemic treatment [1]. Immune activating agents including interleukin-2 (HD IL-2), anti-Cytotoxic T-lymphocyte associated protein (CTLA-4), programmed cell death (PD-1) and programmed death-ligand 1 (PD-L1) antagonists are the most active treatments for many patients with metastatic melanoma [2].

Activation or expansion of immune responses with cytokines or immune checkpoint inhibitors could in theory precipitate GVHD in patients who have undergone ASCT, or dependent on context and timing, could cause rejection of the allograft. In mouse models, anti-CTLA-4 increased lethal GVHD if administered early post ASCT, but the effect on GVHD was diminished if given late after marrow engraftment [3]. Blockade of PD-1 or PD-L1 early post ASCT also increased GVHD lethality in murine models, by mechanisms that appeared distinct from anti-CTLA-4 [4]. We conducted a literature review to determine if GVHD would develop in patients with previous ASCT undergoing therapy with HD IL-2 or anti-CTLA-4 (ipilimumab or tremelimumab) by searching MEDLINE, EMBASE, and Scopus for MeSH terms such as “Melanoma”, “Interleukin-2”, “Graft vs Host Disease”, “Bone Marrow Transplantation”, “Hematopoeitic Stem Cell Transplantation”, “Stem Cell Transplantation”, “CTLA-4 Antigen”, and “Programmed Cell Death 1 Receptor**”.** In a single recent trial of single dose ipilimumab administration after ASCT in 29 patients with relapsing malignancies, an increase in GHVD was not observed [5,6]. No studies were found which reported the safety of these agents in patients developing solid tumors post ASCT with or without prior GVHD. We report the consequences of administering HD IL-2, ipilimumab, and anti-PD1 therapy sequentially to one patient with metastatic melanoma occurring remotely following ASCT, and HD IL-2 alone to a second patient. Neither patient developed evidence of GVHD.

## Case Presentation

### Case 1

A 54-year-old male was treated for Chronic Myeloid Leukemia (CML) in 1985 with homoharringtonine and achieved a complete remission. In 1993, the disease recurred and evolved to a blast crisis. He received total body irradiation and induction chemotherapy, followed by a matched unrelated ASCT. Six weeks after the transplant, he developed a rash on the bilateral hands and abdomen. GVHD was confirmed by biopsy and was treated successfully with steroids and cyclosporine. He received a total of 6 months of cyclosporine, complicated by the development of L3 and L4 osteomyelitis that resolved with antibiotics. He was then followed without further evidence of GVHD.

In 2011, a 5.6 mm deep ulcerated melanoma with 9 mitoses/mm^2^ was resected from the left scapular region. Surgical management included wide excision, sentinel lymph node biopsy and completion axillary lymphadenectomy. One of 11 lymph nodes was positive for melanoma, and the final stage was T4bN1aM0 IIIB. A BRAF V600E mutation was not detected in the tumor. Computed Tomography (CT) of the chest, abdomen, and pelvis in 2011 revealed no evidence of metastatic disease. He was monitored for recurrence until May 2013 when CT scans showed stable small lung nodules and new subcarinal and paratracheal lymphadenopathy. Biopsy of the subcarinal nodes in August 2013 confirmed metastatic melanoma. Over the next several months he began to develop vitiligo in the upper extremities.

After discussion of risks and benefits, he was treated with a version of high dose IL-2, 720,000 IU/kg twice daily on days 1–5 and 15–19, beginning Oct 2013. He received a total of 16 doses. Treatment was complicated by hypotension responsive to IV fluids. CT scans of chest/abdomen and pelvis showed a mixed response, with reduction in size of subcarinal nodes but growth of right mediastinal nodes. An MRI brain also showed several new metastases. Ipilimumab 3 mg/kg q3w x 4 was administered beginning in December 2013, and after the first dose, 10 brain metastases were treated with gamma knife radiosurgery (GKRS). After the second cycle of ipilimumab, he developed a seizure and mild aphasia. MRI of brain showed edema around previously treated lesions and several new lesions. In January, he received GKRS to 8 additional brain lesions. After cycle 3 of ipilimumab, a grade 1 skin rash formed on the skin of the abdomen, legs, and arms that responded to an over-the-counter moisturizer. After cycle 4 of ipilimumab, bevacizumab and celebrex were administered for 3 months to manage the vasogenic edema and mass effect of CNS lesions. In February 2014, CT chest/abdomen/pelvis showed mild progression in chest adenopathy. CT scans and MRI of brain in March 2014 were stable. In late May 2014, repeat CT scans of C/A/P showed mild disease progression in chest adenopathy and 2–3 small new soft tissue lesions. He required GKRS to three new brain lesions in June 2014. During this period of time, he developed progressive and extensive vitiligo. Treatment with pembrolizumab (anti-PD-1) 2 mg/kg IV began on June 19, 2014. Mild chills and fatigue developed in the first 36 hours post-dose but symptoms resolved spontaneously. The 7/28/14 CT C/A/P showed that one mediastinal node was slightly larger but others were smaller and appeared more centrally necrotic. Restaging scans on 11/10/2014 also showed stable disease. Scans on 1/10/15 after 10 cycles of pembrolizumab showed mild progression in chest adenopathy and small retroperitoneal soft tissue nodules. During the course of therapy there was no evidence of severe or ongoing GVHD and the reported rash was more characteristic of ipilimumab. ECOG performance status was maintained at 0 throughout, and he remained asymptomatic except for a mild aphasia.

### Case 2

A 66-year-old male presented in November 2000 with pancytopenia, abdominal lymphadenopathy, and splenomegaly. A bone marrow biopsy was consistent with non-Hodgkin’s lymphoma. After six cycles of CHOP chemotherapy, he underwent modified intensity allogeneic stem cell transplantation in 2002 from his HLA-matched brother. The conditioning regimen included busulfan, fludarabine, and anti-thymocyte globulin. He received tacrolimus and methotrexate for GVHD prophylaxis. Chronic GVHD of the liver was treated successfully with ongoing tacrolimus therapy which was discontinued in 2006.

In 2008, he developed axillary lymphadenopathy diagnosed by biopsy as an angiosarcoma. He was treated with taxol and bevacizumab. Biopsy of a new liver lesion showed metastatic melanoma. Slides of both axilla and liver biopsies were reviewed and the presence of two separate malignancies was confirmed.

In 2009, an MRI of brain obtained for new headache demonstrated a new occipital lobe mass with surrounding edema. The mass was resected with incomplete margins, and the pathology confirmed metastatic melanoma. He was treated with high dose interleukin-2, 720,000 IU/kg twice daily on days 1–15 and 15–19, and received a total of 11 doses limited by hypotension and altered mental status. He also developed erythema of the skin and pruritus that resolved quickly with an over-the-counter moisturizer. One month after completing IL-2, he again presented with more brain metastases and was treated with GKRS and temozolomide. He developed an intracranial hemorrhage and was ultimately discharged to hospice for worsening CNS disease. At no time was there any evidence for GVHD.

## Conclusions

Although rare, solid tumors including metastatic melanoma can develop in long-term survivors of ASCT. Optimal treatment of metastatic melanoma in the post-ASCT setting is not established. Standard therapy for metastatic melanoma has been evolving over the past several years based on improvements in overall survival provided by the immune checkpoint inhibitors ipilimumab, pembrolizumab and nivolumab, and by agents such as vemurafenib, dabrafenib and trametinib in patients with a tumor BRAF mutation. The newer therapies for melanoma are superior to cytotoxic chemotherapy. For select patients, high dose interleukin-2 remains an option for treatment because of its ability to induce durable complete remissions in a small subset of patients [7].

Agents that enhance immune activation, and in particular those like the immune checkpoint inhibitors that induce adverse events resembling GVHD, could theoretically induce or re-activate GVHD in patients with prior ASCT. Because of the latter risk and lack of safety data, clinicians may be reluctant to offer potentially effective immune therapy agents to patients with a history of prior ASCT. In our two patients with metastatic melanoma who had received an ASCT remotely, one received high dose IL-2 alone, and the other high dose IL-2, ipilimumab, and anti-PD1 therapy sequentially. Neither developed GVHD during the course of treatment for melanoma, and despite a prior history of GVHD in patient #1. A mild rash that was typical for ipilimumab toxicity was observed for patient #1 but it was self-limited and responded to an over-the-counter moisturizer [8].

The interval between immune therapy and the ASCT was 20 years and 7 years respectively in the two cases. The long interval between stem cell transplant and immunotherapy may have reduced the risk for developing GVHD [9,10]. The latter observation was consistent with the animal studies of Blazar et al., suggesting that administration of anti-CTLA-4 in the late post-transplant period did not induce significant GVHD. Although less data are available for blockade of PD-1/PD-L1 in the late transplant period, induction of GVHD would not be expected in the absence of active tissue inflammation. Our patient received at least 10 cycles of anti-PD1 without significant toxicity. Because we did not document donor origin of hematopoietic cells in either patient, the lack of GVHD from the immune therapies could also be attributed to re-establishment of host marrow production, although it is likely that both patients’ immune systems were of donor origin.

The adverse effects of the immune checkpoint inhibitors are similar to the manifestations of GVHD, and treatment is similar, although the toxicity of immune therapies is almost invariably self-limited and generally does not require prolonged immune suppression [2,8]. An additional concern is the possibility of a host versus graft rejection, however this would be unlikely in the absence of ongoing mixed hematopoietic chimerism in the recipient.

The lack of significant anti-tumor activity in our patients from IL-2 or anti-CTLA-4 is not surprising in the absence of an effect on GVHD. Nevertheless, the minor activity observed from anti-PD1 in one patient suggests that anti-tumor T cell responses can form in patients with prior ASCT, and these responses may be inhibited by one or more immune suppressive mechanisms in the tumor microenvironment. Our experience, although limited, would support consideration of cytokines or immune checkpoint inhibitors as a treatment option in patients with a remote history of ASCT. Assuming the existence of host determinants that could be recognized by donor lymphocytes, it is remarkable that we did not observe GVHD, and indicates the degree of tolerance that forms over time. Similar to otherwise ‘normal’ patients, other checkpoint inhibitors, or combinations of checkpoint inhibitors with each other or with co-stimulatory agents, may break tolerance and produce greater immune related adverse events. However, the risk-benefit ratio may also change if the treatment is also associated with increased efficacy. The rarity of these patients precludes systematic study, but careful evaluation in individual patients may provide important precedents for treatment of future patients with similar agents.

## Consent

Written informed consent was obtained from the patient for publication of this Case report and any accompanying images. A copy of the written consent is available for review by the Editor-In-Chief of this journal. For the deceased patient above, next of kin could not be contacted and special permission by the Editor-in-Chief was granted to include the patient in the series without a signed consent.

## Abbreviations

GVHD: Graft versus host disease
IL-2: Interleukin-2
CTLA-4: Cytotoxic T-lymphocyte associated protein
PD-1: Programmed cell death
PD-L1: Programmed death-ligand 1
ASCT: Allogeneic stem cell transplants
CML: Chronic Myeloid Leukemia
CT: Computed Tomography
GKRS: Gamma knife radiosurgery
